# Identification of Controlled-Complexity Thermal Therapy Models Derived from Magnetic Resonance Thermometry Images

**DOI:** 10.1371/journal.pone.0026830

**Published:** 2011-11-02

**Authors:** Ran Niu, Mikhail Skliar

**Affiliations:** 1 Department of Chemical Engineering, University of Utah, Salt Lake City, Utah, United States of America; 2 Department of Chemical Engineering, University of Utah, Salt Lake City, Utah, United States of America; University of California, Berkeley, United States of America

## Abstract

Medical imaging provides information valuable in diagnosis, planning, and control of therapies. In this paper, we develop a method that uses a specific type of imaging—the magnetic resonance thermometry—to identify accurate and computationally efficient site and patient-specific computer models for thermal therapies, such as focused ultrasound surgery, hyperthermia, and thermally triggered targeted drug delivery. The developed method uses a sequence of acquired MR thermometry images to identify a treatment model describing the deposition and dissipation of thermal energy in tissues. The proper orthogonal decomposition of thermal images is first used to identify a set of empirical eigenfunctions, which captures spatial correlations in the thermal response of tissues. Using the reduced subset of eigenfunction as a functional basis, low-dimensional thermal response and the ultrasound specific absorption rate models are then identified. Once identified, the treatment models can be used to plan, optimize, and control the treatment. The developed approach is validated experimentally using the results of MR thermal imaging of a tissue phantom during focused ultrasound sonication. The validation demonstrates that our approach produces accurate low-dimensional treatment models and provides a convenient tool for balancing the accuracy of model predictions and the computational complexity of the treatment models.

## Introduction

In ultrasound (US) thermal therapies, the goal is to selectively heat the treatment target without excessively elevating the temperature in healthy tissues intervening in the path of transmitted US energy and surrounding the target. In recent years, temperature measurements obtained by MR thermometry has played an increasingly important role in planning and control of thermal therapies. A number of techniques have been developed to obtain MR temperature maps, with water proton resonance frequency (PRF) method being the most widely used in practice [1]. Several studies [2]–[4] have demonstrated that MR-thermometry can be used as a feedback in automatic treatment control systems. If we view images as a collection of pointwise measurements associated with each pixel and use these data to obtain models to plan, optimize and control therapies, the dimension of the resulting models will be high. For example, the pointwise utilization of 512
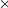
512 voxels in MR thermal images would lead to a treatment model with over 250,000 states. This presents a problem of a very high computational demand when a model must be used in real time during the therapy, as in the case of a model-based and optimizing treatment control systems, such as the one described in [4].

In this paper, we develop and validate methods that avoid pointwise utilization of imaging data, leading to a highly efficient compression of MR thermometry images and the identification of a low-dimensional dynamic treatment models. Fundamentally, a low-dimensional representation of MR thermal images is possible because the image voxels are spatially correlated, reflecting the dependence of temperature distribution in the treatment target on the specific absorption rate (SAR) of the ultrasound and the heat dissipation by conduction and convection. Furthermore, a time series of MR thermal images are correlated temporally because of the causal dependance of temperatures on the heating history and temperatures at the preceding times. In the developed approach, we apply proper orthogonal decomposition (POD) to identify orthonormal basis functions 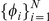 which capture spatial correlations in the ensemble of 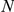 MR thermal images. Each element 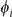 is identified to capture the maximum amount of spatial correlations that have not yet been explained by a subset of previously identified basis elements. We then select the reduced basis 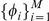, 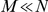, consisting of the first 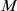 basis elements, such that the desired balance between accuracy and complexity is obtained. The selected reduced basis is then used to:

- Approximate each image acquired during therapy in the reduced set of basis functions. This step may be viewed as a real time image compression, which exploits spatial correlations of image voxels, as well as the image filtering step since high-frequency spatial valuations, which usually correspond to MRI measurement noise, are removed.
- Identify the ultrasound specific absorption rate and the dynamic model that captures temporal correlation in the series of images and provides a prediction the evolution of the ultrasound treatment.

The developed approach is validated experimentally, using an *in vitro* MRI tissue phantom heated by a focused ultrasound transducer. The results show that the identified low-dimensional models predict the SAR and the temperature response of the target with the expected accuracy, which can be controlled by selecting the order 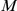. Even with a very low-dimensional model, suitable for use in model-predictive treatment control systems, thermal images in the verification set were accurately approximated and the predictions of the SAR and the phantom's thermal response closely agreed with the MRI measurements.

## Methods

### Identification of basis functions using POD method

The proper orthogonal decomposition is a technique often used to extract a set of basis functions for an approximate, modal-like representation of an infinite-dimensional, distributed parameter system (DPS). It was previously shown [5] that POD is the most efficient way to obtain dominant modes of a *known* infinite-dimensional dynamic system, which makes it a popular approach in a variety of applications, including image processing [6]. Often, a numerical solution of a given partial differential model at different times (known as snapshots) is used as the input to the POD algorithm to produce the desired basis functions. The identified basis is then used in combination with the projection methods to obtain a finite-dimensional approximation of the original known infinite-dimensional DPS model [7].

In this paper, we use a time series of images to identify an orthonormal basis of an *unknown* infinite-dimensional model, characterizing the heat transport in the target due to noninvasive ultrasound heating. The following brief outline gives the exposition to the POD method in the context of our objectives. Further theoretical and algorithmic details are available in [5], [7]–[9].

Consider a set of MR thermal images (snapshots) 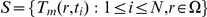. Here, 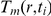 is an image of the region of interest (ROI), characterized by a vector of coordinates 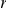 inside the spatial domain 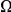 and acquired at time 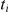. The problem is to obtain a function 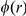, which is the best at capturing the spatial distribution of temperatures in the ensemble 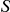 of snapshots 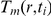. Mathematically, the problem is to find 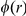, such that the projections of all snapshots 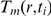 onto 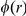 are maximized:
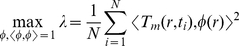
(1)where 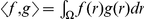 denotes the inner product of square integrable functions 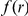 and 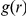 defined in 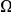. The normalization condition 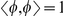 is imposed to ensure uniqueness of the solution.

The optimization problem (1) is difficult to solve in the general case. The problem is simplified if, following [10], we seek the solution under an additional assumption that 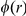 can be expressed as a linear combination of snapshots:
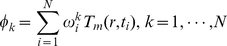
(2)and all basis functions 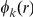 are orthonormal:
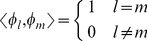
(3)In this case, it can be shown [11] that the solution of the optimization problem is reduced to the solution of the following matrix eigenproblem:
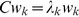
(4)where 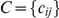 is the covariance matrix of all snapshots with elements
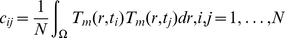
(5)For a strictly positive definite matrix 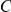, equation (4) is satisfied by 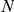 orthogonal eigenvectors 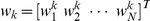 of dimension 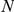 and the corresponding distinct eigenvalues 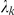, where 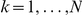. The elements 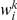 of an eigenvector 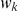 are the coefficients in the sought linear decomposition (2), and thus determine the 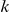-th eigenfunction 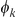. The requirement that 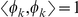 of problem (1) is enforced by normalizing all eigenvectors, such that 
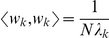
.

Assuming that all eigenvalues are ordered (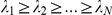), the eigenfunction 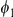, corresponding to 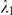, is the desired solution 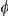 of the maximization problem (1). The normalized eigenfunctions 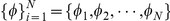 form an orthonormal basis of the image ensemble, 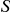. The amount of information, captured by the projection of 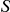 on the 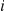-th eigenfunction, is characterized by the corresponding eigenvalue:
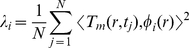
(6)Consequently, 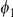 is the best at explaining spatial correlations in 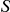, followed by 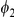 as the next most informative direction, and so on.

When the complete set of the identified eigenfunctions is used to represent images in 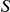, there is no loss of information. There is no benefit either since the basis order 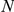 is equal to the number of images in 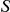. The question is how to select a reduced basis of order 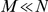 to obtain the desired accuracy of the image approximation in the reduced basis. To answer this question, we start by defining the “energy” of an image ensemble as
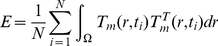
(7)It is easy to show that
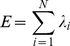
(8)Therefore, we can use the eigenvalues to guide the selection of the reduced-order POD basis. One approach is to select 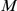 such that a predetermined fraction 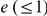 of the total energy of the ensemble 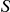 is captured. Specifically, we may wish to select the smallest 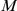 such that
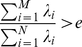
(9)where 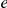 is selected by a user. The reduced-order basis 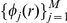 can then be used to parsimoniously approximate all images in the ensemble 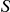, as well as all new images acquired during ultrasound treatment. In the sequel, this reduced basis is also used to identify low-dimensional patient- and site-specific ultrasound SAR and thermal treatment models based on the information captured by MR thermometry images.

### Image representation in the reduced basis

Consider a thermal image 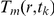, collected at time 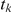, which reflects the spatial temperature distribution in the region of interest 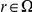 in response to the sonication history over the time 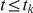. Thermal effect of sonication is described by the power deposition, 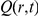, which relates to the ultrasound specific adsorption rate (in W/kg) as
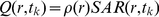
(10)The problem for approximating a new image can be viewed as the minimization problem of finding projections 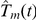 of an image 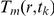 into the manifold, spanned by a reduced basis of empirical eigenfunctions 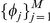:
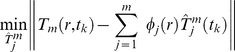
(11)The solution of (11) is reduced to finding the best solution (in an appropriately selected norm) of the following equation:
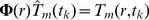
(12)where 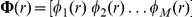 is a row vector of eigenfunction. We will now take into account that 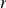 takes only discrete values in 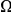 due to image pixelation (finite spatial resolution). Therefore, each basis function, formed according to equation (2), is given by its values in the spatial locations of image voxels, or 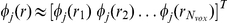, where 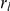 is the coordinate of 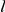-th voxel and 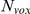 is the total number of voxels. With finite spatial resolution, a vector of function 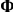 becomes the 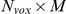 matrix, which transforms (12) into a system of 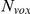 linear equations in 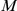 unknown, and by solving (12) in the least squares sense, the projection vector 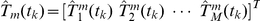 is obtained. Note that the repeated solution of (12) for each acquired image can be accelerated by parameterizing matrix 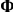 (for example, by calculating its 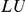 decomposition).

With the described procedure, the temperature measurements 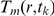 are completely encoded, to the desired accuracy, by a low-dimensional vector of projections 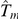 of dimension 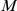, where 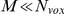. Note that only projections 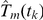 must now be sent from the MRI scanner, which reduces communication traffic. The storage requirements are also reduced because only a 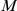-dimensional vector of projections must be saved for each newly acquired thermal image of 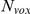 (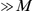) voxels.

Our previous results [11] indicate that by ignoring the contribution of higher order eigenfunctions 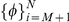 in image representation spatial noises in MR thermometry images are filtered.

### Representation of ultrasound power deposition

The temperature distribution measured by MR thermometry depend on the power deposition in tissue, 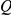 (in W

m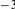), caused by sonication. Therefore, it is reasonable to expect that 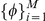, identified using the acquired images, is also an adequate basis to represent 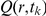. Similarly to equation (12), for known 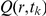, the projection of the power deposition into the reduced basis, 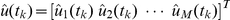, can be found as the least squares solution of the following linear equation:
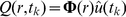
(13)


### Identification of the projection models of thermal response

**Figure 1:**
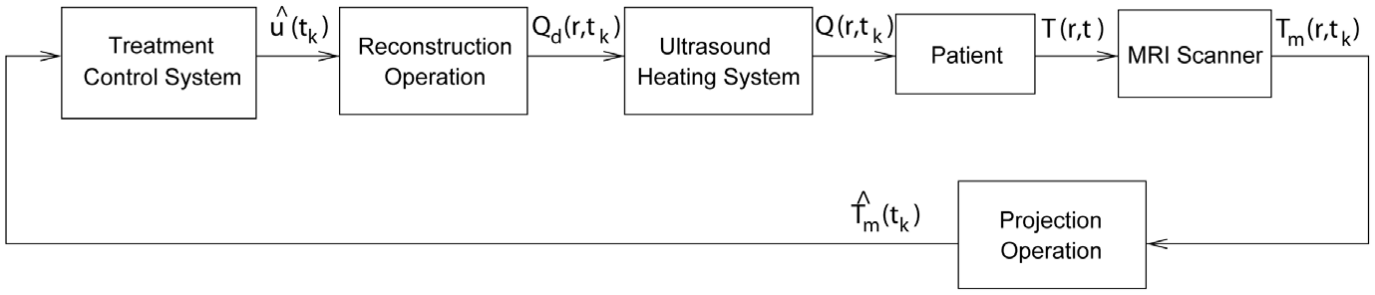
Treatment control system uses low-dimensional projection models of the therapy and the SAR.

Our objective is to to identify an ultrasound thermal treatment model in a low-dimensional projection form with the state vector, 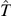, corresponding to the projection of measurements, 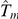. A low-dimensionality is required to enable real time utilization of treatment models for such tasks as intra-operative prediction of specific sonication parameters on temperature distribution and to design efficient model-based treatment controllers. Figure 1 shows a block diagram of a control system that uses projections 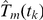 (instead of full-dimensional images, 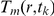) in its feedback and determines control inputs, 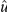, using low-dimensional treatment model in projection form. This figure illustrates that during ultrasound therapy the images from the MRI scanner are sent to the control system in low-dimensional form of image projections 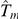, and the controller uses this information to generate 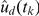, which describes 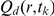 – the desired power deposition which we wish to apply to the patient at the current time; 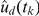 and the corresponding 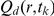 are the control inputs sent to the ultrasound subsystem. The best approximation of 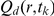 implementable with the available ultrasound transducers or transducer array system, 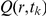, is then applied to the patient. As desired, the described control system entirely avoids high-dimensional (pointwise) computations.

We seek to identify the low-dimensional treatment model in the following form:
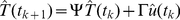
(14)where 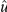 is the projection of the ultrasound power deposition, 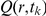; matrices 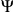 and 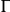 are unknown, and must be identified from the images 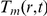 in the training ensemble, 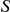. The state vector 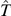 is the predicted temperature projection; given 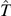, the predictions of the temperature distribution in the region of interest (ROI) can be obtained as
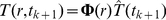
(15)


It is straight forward to show that all linear PDE models, traditionally used to describe temperature evolution in biological tissues during thermal treatments (such as the convection-diffusion model and its different approximations), can be adequately approximated by model (14). Consider, for example, the following popular Pennes' bioheat transfer equation (BHTE) [12], which is written in terms of the temperature increase 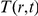 above the equilibrium:
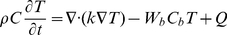
(16)where 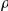 (kg/m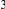) and 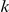 (W/m

C) are the tissue density and thermal conductivity, 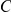 and 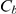 are the specific heat of tissue and blood (in J/kg

C), respectively. Equation (16) does not require the detailed information on tissue vascularity or blood flow but instead uses an empirical blood perfusion-related parameter 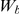 (kg/m
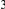
s). After using 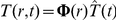 and 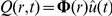 in equation (16), and approximating time derivative using backward-difference approximation, the BHTE takes the following form:
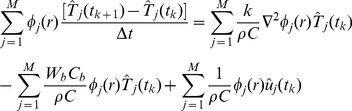
(17)where 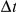 is the time discretization step. The weak Galerkin formulation of the Pennes' model is obtained by taking the inner product of equation (17) with the elements 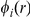 of the reduced basis, yielding the following system of discrete-time dynamic equations:
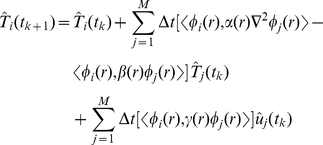
(18)where 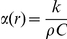, 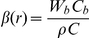, 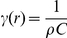 and we took into the account the orthonormality of the basis functions. The compact form of the 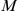-dimensional projection model (18) is exactly the same as equation (14) with matrices 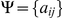, 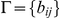 defined by their elements as:
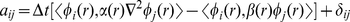
(19)

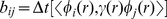
(20)where 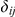 is the Kronecker-delta, and 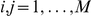. Note that for a constant tissue density and heat capacity, 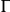 is a diagonal matrix 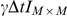.

Matrix 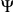 characterizes the heat dissipation in the target due to conduction and blood flow, while the affine term 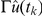 describes the effect of the ultrasound power deposition 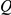 on the evolution of projected temperature responses 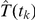.

The problem is to identify 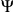, 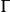 and 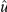 such that projections 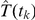, predicted by the model (14) and used to reconstruct temperature distribution as 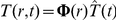, give the best agreement with the acquired series of images 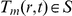.

#### Identification of matrix 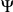


In order to decouple the problems of identifying 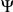, 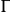 and 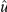, the system matrix 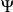 is identified first by utilizing only thermal images 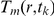 acquired during tissue cooling (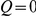) to thermal equilibrium. With zero power input 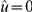, the projection model (14) describes the decay of temperature projections to thermal equilibrium 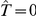 from non-zero initial conditions:
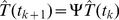
(21)This equation is in the form of a first-order multivariate autoregressive (AR) model, which allows us to identify 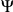 using the standard system identification techniques [13].

#### Identification of the affine term

The specific absorption rate of an ultrasound transducer depends on tissue properties and other variable factors, which can change with treatment site and from patient to patient. At the same time, the knowledge of the patient- and site-specific SAR is critically important for treatment safety and its precise control.

Within the developed approach, the identification of the SAR is accomplished easily. Once the system matrix 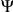 is determined, the time-varying affine term 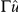 can be identified at each sampling instant 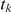 as:
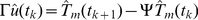
(22)where 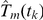 and 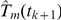 are the projections of the consecutive thermal images, collected during sonication of the target. Since 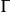 (cf. equation 20) depends only on the MRI scan time 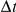, the identified eigenfunctions, and the relatively little-changing tissue density and heat capacity (both often assumed equal to the water values), the matrix 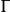 in the projection model can be considered as known. With this assumption, 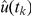 can be found as a least squares solution of the linear equation (22). The corresponding estimates of 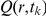 and the SAR then be obtained by using equations (13) and (10).

## Results

**Figure 2:**
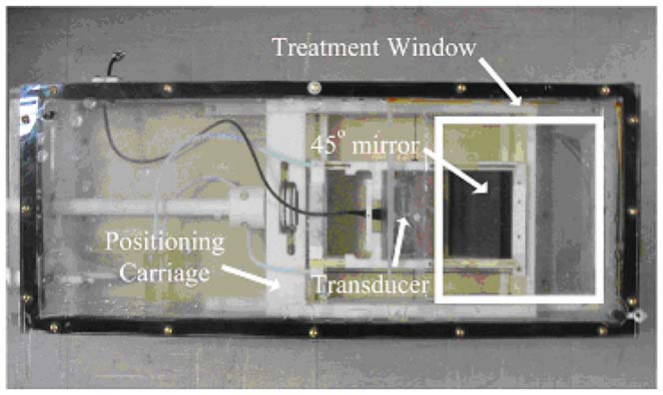
MR-compatible ultrasound transducer and positioning system.

The developed approach to identifying low-dimensional models of ultrasound therapies was tested experimentally using MR measurements of the thermal response of a tissue phantom to focused ultrasound (FUS) sonication. The cubic 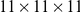 cm phantom was prepared following the recipe of Madsen *et al.* [14]. The T2 relaxation time of the phantom was modified by adding one millimole-per-liter of copper sulfate to the recipe. After preparation, the phantom was allowed to solidify inside of an acrylic box with a Mylar ultrasound treatment window on the bottom surface. The ultrasound power deposition field was created by a single, spherically focused, air backed ultrasound transducer with aperture diameter of 10 cm and radius of curvature of approximately 18 cm, and resonating frequency of 1.5 MHz. The transducer was placed in a bath of degassed and deionized water inside the MR compatible ultrasound positioning system (Figure 2 and [4]), which was designed to move the focal zone in three dimensions. After initial alignment, the transducer position remained fixed in the current experiments. The transducer was driven by a function generator (Stanford Research System, model DS345), amplified using RF amplifier (ENI, model A150). The electrical impedance of the transducer was matched to the output impedance of the amplifier using an external LC matching circuit. The electrical power applied to the transducer (direct and reflected) was measured using power meters (Hewlett-Packard, model 435A/B). Further details of driving circuitry and positioning system are given in [15].

Temperature increase, 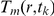, inside the phantom was measured using Siemens Trio 3T Magnetom scanner. A custom receive-only surface coil was used to improve the temporal and spatial resolution of the acquired thermal images. The coil created a localized sensitivity pattern, which minimized the interferences and improved the signal-to-noise ratio (SNR). Gradient-echo sequence (GRE) with the following acquisition parameters was used to obtain temperature measurements: repetition time 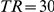 ms, echo time 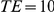 ms, field of view 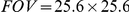 cm and flip angle 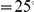. The voxel size of thermal images was 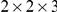 mm. The scan time was 2.45 s with the phase resolution of fifty percent to increase the sampling rate. The overall image size was 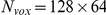 and the 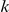-space data were zero-filled to form a 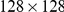 data matrix.

Following the PRF shift method [16], the phase difference 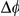 between the two consecutive phase images was used to calculate the relative temperature change 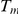 as
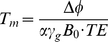
(23)where 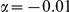 ppm 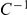 [17] is the coefficient of PRF shift for aqueous tissue, 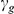 is the gyromagnetic ratio, 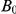 is the strength of the main magnetic field, and 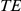 is the echo time. Sequential complex MR images 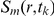 and 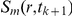 were used to calculate 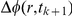 as the phase of the following product:
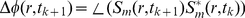
(24)where 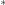 denotes the complex conjugate operator.

**Figure 3:**
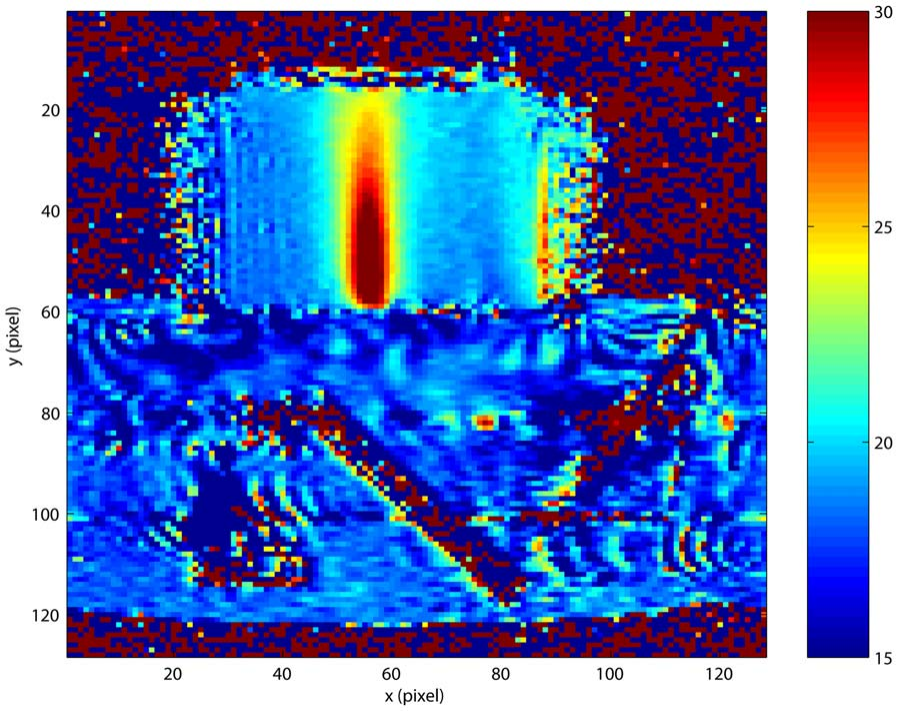
Coronal MRI map of temperature elevations inside the phantom heated by focused ultrasound transducer.

Figure 3 gives a representative temperature image in the US transducer's focal plane (gradient scale is in 
C). The phantom appears as a rectangular object above the ultrasound positioning system containing a clearly visible 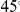 ultrasound mirror. The selected ROI (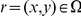) is the region of an appreciable temperature elevation, which has pixel coordinates 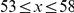 and 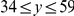. The number of voxels in the ROI is 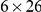; its actual dimension is 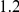 cm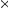 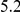 cm. As expected, the maximum temperature rise is observed on the line of focal symmetry, 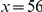.

### Validation Results

A step increase from zero to either 3.5, 4.8 or 6.5W of total electrical power (direct minus reflected) was applied to the FUS transducer while keeping all other experimental conditions the same. The phantom was allowed to reach thermal equilibrium before changing the power input.

MRI thermal images collected for the case of 6.5W of applied electrical power were used as a *training (estimation)* dataset to identify the reduced-order basis, and the corresponding models of the thermal response and the ultrasound SAR. The *validation* datasets, consisting of the images collected during the experiments with the other two power levels, were used to assess the adequacy of the identified basis functions and the accuracy of the temperature predictions obtained with the identified ultrasound treatment model.

**Figure 4:**
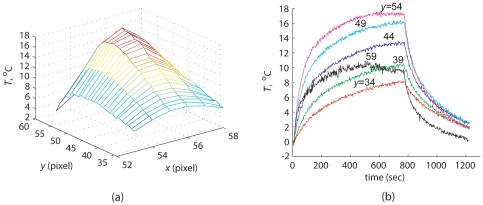
Model estimation data set. (a) MRI measurements of temperature increase in the ROI at 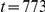 s. (b) Temporal evolution of temperature increase on the line of focal symmetry 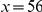 measured by MR thermometry.

A total of 499 MR thermal images were acquired to characterize temperature evolution during each power step test. The estimation dataset included images collected when the power was kept constant at 6.5W (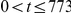 seconds) and when the tissue was cooling back to equilibrium (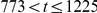) after the power was switched off. Figure 4(a) shows the measured temperature elevation within the ROI at 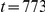 s when the temperature reaches its peak value. The temporal evolution of temperatures in the selected locations on the line of ultrasound beam symmetry is shown in Figure 4(b). The highest temperature was observed at 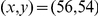, where the maximum temperature increase from ambient temperature was 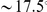
C.

#### Identification of the reduced-order basis

**Figure 5:**
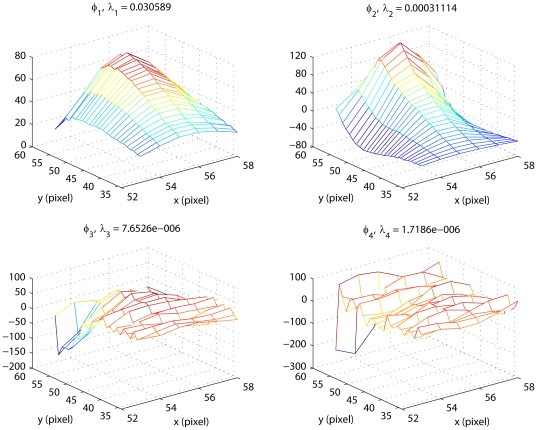
Identified eigenfunctions for the ROI.

All 499 images in the estimation dataset were used to identify the orthonormal basis following the described method. Figure 5 shows the first four identified eigenfunctions and the corresponding eigenvalues, which rapidly decay for the higher order 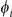
's. Using the criterion (9), it was determined that the first eigenfunction captures approximately 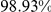 of the spatial correlations in the collection of 499 images, while 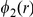 captures only 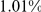. Selecting 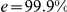, we conclude that high accuracy of image approximations in the reduced POD basis 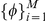 is achieved with only two basis functions (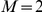). Note that the shape of 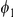, identified to maximize the explained spatial correlations in thermal images, is similar to the shape of temperature distribution, as expected. Further note that eigenfunctions 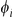, 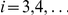 capture information at increasingly higher spatial frequencies, and that ignoring their contribution in image representation (15) has the effect of a spatial filtering of imaging data.

#### Identification of thermal response and SAR models

Thermal images, collected during tissue cooling after the initial sonication at 6.5W of constant applied power, were used to identify the system matrix 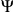 of the projection model (14). The result of Table 1 was obtained by, first, using Matlab's System Identification Toolbox [13] to obtain the autoregressive model of the cooling process and then converting it into the state space form of equation (21).

**Table 1:** The identified SAR and the thermal response model.

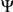	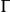	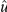
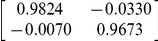	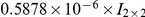	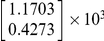

Handbook values of heat capacity and density (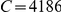 J/(kg

**Figure 6:**
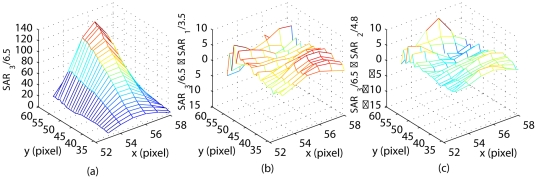
Model identification results. (a) The identified SAR pattern for 6.5W of the applied power. The result is shown after pointwise scaling of SAR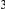 with 6.5. (b) The prediction error of the scaled SAR pattern for 3.5W of applied power. (c) The prediction error of the scaled SAR pattern for 4.8W of applied power.

C and 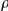 = 1000 kg/m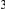) [18]) were used to calculate 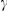 in equation (20). Sequential thermal images were used in equation (22) to estimate the power deposition projection 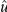. The identified vector 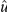 (Table 1) was then used in equations (13) to estimate 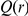; the corresponding 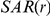 was obtained using equation (10). The result (SAR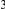), scaled with the total applied power of 6.5W, is shown in Figure 6(a). A high degree of correlation between the shapes of the measured temperature distribution (Figure 4a) and the SAR is an expected result for the unperfused phantom.

The prediction of the thermal response to different power inputs is based on the assumption that the shape of the SAR remains constant for the fixed relative transducer-patient position, but the SAR values are scaled with the applied electrical power. This assumption of the linear SAR-power dependence was tested by comparing the predicted SAR pattern for 3.5 and 4.8W of applied power, obtained by scaling SAR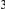 by 3.5/6.5 and 4.8/6.5, with the directly identified SAR distribution (SAR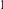 and SAR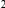). The direct identification of SAR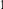 and SAR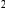 followed the same method used to identify SAR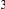 (i.e. a new reduced POD basis and the corresponding thermal model were obtained in each case; equation (22) was used to calculate a new 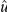; then SAR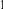 and SAR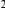 were reconstructed using equations (13) and (10)).

Figures 6(b) and (c) shows the difference between the predicted and the directly identified SAR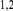, both scaled to 1W of the applied power. The maximum pointwise absolute prediction errors are 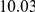 and 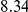 (in W/kg), respectively. Low prediction errors confirm that in our experiments the specific absorption rate changed linearly with the applied power.

#### Validation of the thermal response model

The accuracy of the identified reduced-order treatment model was assessed in projection manifold and in terms of temperature predication errors. In projection space, the prediction of the temperature projection vector 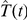, obtained with the same two-dimensional state space model of Table 1 and the appropriately scaled 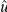, was compared with the projection of the actual thermal images 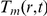, acquired during the experiments with three different power levels. Image projections, 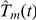, were found as the least squares solution of equation (12).

**Figure 7:**
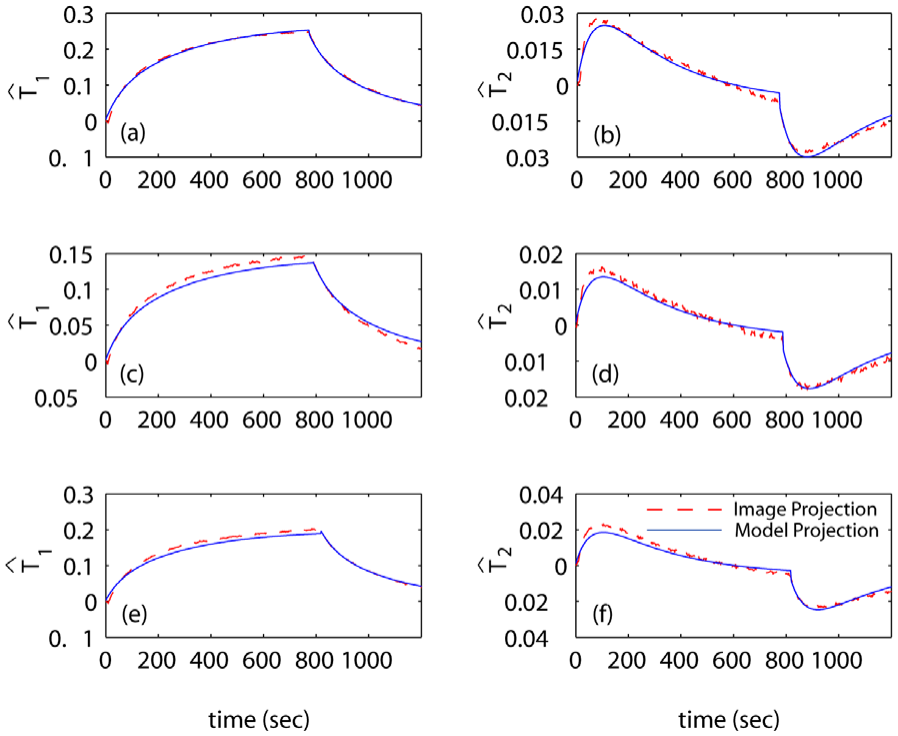
Comparison of measures and model predicted temperatures in projected form. The projections, 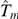, of the acquired images are compared with model predictions, 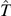, obtained with the identified thermal response model. The value of 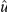 listed in Table 1 was used to make predictions for the case of 6.5W of applied power (plots (a) and (b)). Predictions for the power inputs of 3.5W (plots (c), (d)) and 4.8W are made by scaling 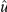 by 3.5/6.5 and 4.8/6.5, respectively.

Figure 7 shows an excellent agreement between the predictions, 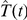, and measurements, 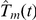. The agreement is the best for 6.5W of applied power – the case used as the estimation dataset. The plot of 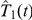 is similar in shape to the pointwise temperature evolutions (cf. Figure 4b), which indicates that the first component of the vector 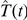 captures most of the slow temporal variations in the series of thermal images.

**Figure 8:**
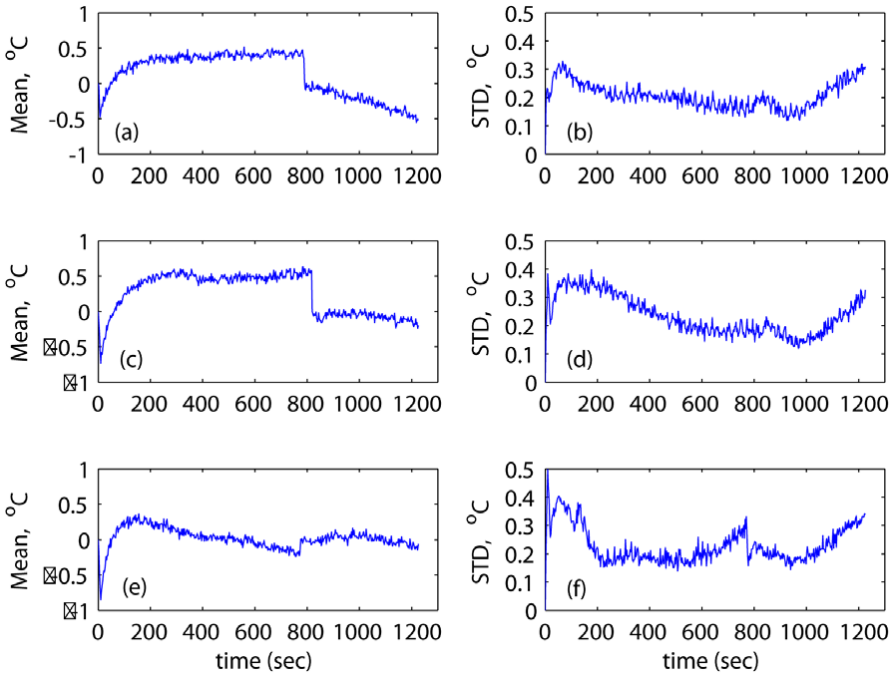
Spatial mean and standard deviation of the prediction errors. Graphs (a)–(b) show the results for 3.5W of applied power; graphs (c)–(d) and (e)–(f) are for the case of 4.8W and 6.5W of applied power, respectively.

When used in equation (15), the model-generated 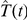 gives the prediction of the temperature elevation, 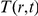, in the ROI, which can be compared with imaging data, 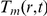. Figure 8 shows the spatial mean and standard deviation (STD) of the temperature prediction errors for all pixels in the ROI. The prediction errors are small, including the two validation cases shown in subplots (a)–(d). The maximum pointwise temperature prediction errors do not exceed 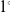
C, which is of the same order as the measurement noise of MRI thermometry.

## Discussion

The developed approach was shown to be effective in identifying low-dimension but accurate models of ultrasound thermal therapies. At a pre-treatment stage, a set of MR thermometry images, characterizing the response of the target and the surrounding normal tissue to thermal excitation, is collected and then used to identify the reduced POD basis, which capture spatial correlations in images. A simple criterion for selecting an appropriate number of basis functions is provided which allows a user to balance the computational complexity of a predictive treatment model with its computational complexity. The selected reduced basis is then used to parsimoniously approximate newly acquired images, thus minimizing storage and data traffic. As an additional benefit, image approximation in the reduced basis filters high-frequency spatial noises in MR images.

The SAR and thermal response models identified following the developed approach are patient- and site-specific and can be used as predictive models in real time (e.g., treatment control) and off line (such as treatment planning) applications. The identification procedures are well suited to perform continuous re-identification of treatment models during the therapy. Such intra-treatment adaptability helps to mitigate the effect of changing tissue properties (such an US absorption) and blood perfusion, caused by elevated temperatures, on the accuracy of model predictions, which is particularly important when the identified low-dimensional models are used to efficiently implement model-based, optimizing treatment controllers that utilize images in the feedback. A family of related results used to identify continuous-time treatment models is described in reference [19].

The developed methods were validated during *in vitro* MR experiments with a tissue phantom heated by a focused ultrasound transducer. The experimental results indicate that the SAR and thermal response during the treatment can be accurately predicted by the identified projection models with only two states. The low-dimensionality of the identified models substantially minimizes computational requirements of implementing a model-based treatment control system and communication traffic between the MRI scanner and the treatment controller.

Though our emphasis is on thermal therapies, the developed approach has a broader applicability in image-based identification and image-guided control of therapies. After straightforward modifications, this approach can be used with images acquired in multiple planes and with three-dimensional MR measurements.
